# Body lice and scabies co-infestation among unsheltered migrants, refugees, and asylum seekers and the right to water and sanitation

**DOI:** 10.1371/journal.pntd.0013807

**Published:** 2025-12-10

**Authors:** Carl Boodman, Wilma van den Boogaard, Guido Benedetti, Federica Zamatto, Azzurra D’incà, Jovana Arsenijević, Tobias Janisch, Temmy Sunyoto, Corey Leclair

**Affiliations:** 1 Clinical Investigator Program, University of Manitoba, Winnipeg, Manitoba, Canada; 2 Department of Clinical Sciences, Institute of Tropical Medicine, Antwerp, Belgium; 3 Department of Medical Sciences, University of Antwerp, Antwerp, Belgium; 4 Luxembourg Operational Research Unit, Médecins Sans Frontières, Luxembourg City, Luxembourg; 5 Médecins Sans Frontières-Operational Centre Brussels, Brussels, Belgium; 6 Médecins Sans Frontières-Operational Centre Brussels, Belgrade, Serbia; Egerton University, KENYA

Body lice (*Pediculus humanus humanus*) and scabies (*Sarcoptes scabiei var. hominis*) proliferate under conditions of overcrowding and poor hygiene [1]. Co-infestation with these ectoparasites remains under-recognized among unsheltered migrants, refugees, and asylum seekers. These diseases are associated with significant morbidity and, in severe cases, mortality due to bacterial complications.

In October 2016, an epidemic of body lice and scabies infection was declared by the Institute of Public Health of Serbia in response to evidence gathered by Médecins sans Frontières (MSF) [2]. Within a few months, thousands of cases of body lice infestation and scabies were documented in Belgrade among migrants, refugees, and asylum seekers using the Western Balkan corridor to reach Northern Europe [3]. Diagnoses were made clinically for scabies and visually for body lice. The target population slept in overcrowded industrial structures with extremely limited access to water, impeding the maintenance of basic personal hygiene. To mitigate human suffering, MSF implemented a targeted intervention to screen, manage, and prevent ectoparasitoses [2]. The program was called “Safai”, meaning “cleaning, rinsing, purifying” in Urdu, Pashtun, and Farsi, the predominant languages of the migrating population [2]. The MSF-Safai program offers enduring insights that are applicable to public health strategies addressing migration-related challenges (Table 1).

**Table 1:** Key lessons from the MSF-Safai program on body lice and scabies co-infestation.

Lesson	Rationale	Implementation
Integrate systematic screening for body lice during scabies outbreaks among migrant populations experiencing homelessness	Without systematic screening of clothing seams, body lice infestations went undiagnosed. Following routine screening in the MSF-Safai program, body lice were identified in the majority of the target population and among most individuals with ectoparasitosis, either alone or co-infested with scabies. Body lice outbreaks may trigger the reemergence of louse-borne diseases (*B. quintana*, *R. prowazekii*, *B. recurrentis*). Lice can also serve as surveillance tools for these pathogens.	Include clothing inspection for adult lice and eggs during scabies outbreak investigations. Train staff in screening, lice collection, and safe disposal. Establish referral links with microbiology laboratories for diagnosing louse-borne disease.
Prioritize screening in overcrowded and high-risk settings	Overcrowding, poor hygiene, and limited access to water and sanitation amplify transmission, especially in informal settlements around migration bottlenecks and border zones.	Focus screening in high-density shelters and camps. Anticipate ectoparasite outbreaks when new migration barriers are introduced. Integrate lice checks into clinical and outreach services targeting at-risk populations (e.g., mobile clinics).
Establish interdisciplinary outbreak response teams	Effective control requires collaboration among medical, environmental, and vector-control professionals, as well as cultural mediators. Each contributes unique expertise in case management, surveillance, environmental control, and communication.	Coordinate with public health authorities, as soon as possible. Define roles clearly and maintain regular coordination meetings.
Strengthen vector-control measures	Entomologist-led insecticide use and environmental decontamination are essential for controlling transmission and re-infestation. Due to the unavailability of other products, the MSF-Safai program applied 0.19% permethrin spray to affected individuals and provided permethrin-impregnated clothing. Benzyl benzoate 25% cream was added for scabies.	Follow expert guidance on insecticide availability, resistance, and use in your jurisdiction. Combine with hygiene interventions.
Collaborate with laboratories for pathogen testing	Body lice transmit *B. quintana*, *B. recurrentis*, and *R. prowazekii*; early detection among collected lice may identify concealed cases of louse-borne disease.	Coordinate with reference laboratories to test collected lice for louse-borne pathogens using PCR.
Improve access to hygiene infrastructure/water and sanitation	Sustained control requires adequate washing, laundry, and clean clothing, which are often lacking in displacement contexts.	Provide showers and laundry access; use temporary solutions (e.g., bucket showers) while establishing permanent systems. Coordinate with public sector engineers, when possible.
Partner with local organizations for clean clothing and bedding	Many migrants lack spare clothing during treatment, undermining delousing efforts.	Work with civil societies, NGOs, charity organizations and local partners to ensure access to clean clothes, bedding, and blankets during treatment and laundering.
Facilitate access to effective treatments, including oral ivermectin	Topical treatments (permethrin, benzyl benzoate) require full-body application while unclothed and are therefore impractical in settings lacking privacy (permethrin is further limited by reports of resistance). Oral ivermectin (2 or 3 doses of 200 µg/kg, 7–14 days apart) is effective and may be more acceptable than topical agents but is often limited in availability or with limited human indication^*^ [10,14].	Upon outbreak detection, engage with public health departments, pharmacies, and NGO partners to advocate for ivermectin human use, procurement and regional imports. Consider mass-drug administration.^*^
Advocate for equitable access to water and sanitation	Access to water and sanitation is a human right and critical for sustained ectoparasite control. Ongoing gaps perpetuate outbreaks among displaced populations. Many countries have endorsed resolutions on the right to water and sanitation [15].	Collaborate with advocacy and legal partners to promote water and sanitation commitments. Highlight ectoparasite outbreaks as indicators of neglect and unmet international obligations.

* For individuals from *Loa loa*–endemic areas, ivermectin should be administered with caution, as severe post-treatment encephalopathy has been reported in cases with high *Loa loa* microfilarial blood concentrations. In many jurisdictions, ivermectin may be limited to veterinary use.

## Body lice infestation

Body louse infestations are caused by the *Pediculus humanus humanus* ecotype of the human louse species *P. humanus* [1]. Unlike head lice which live at the base of hair shafts, body lice live in human clothing. Body lice are the primary vectors of three bacterial pathogens: *Bartonella quintana* (historically, trench fever), *Rickettsia prowazekii* (epidemic typhus), and *Borrelia recurrentis* (louse-borne relapsing fever) [1]. These bacteria are transmitted between human hosts through the feces of infected lice, contaminating abrasions, bite sites, and mucous membranes [1]. *B. quintana* is the most commonly identified pathogen in body lice, present in 19% of body lice infestations globally [4]. Originally described in World War I as the cause of trench fever, in contemporary times, *B. quintana* is associated with infective endocarditis, chronic bacteremia, and bacillary angiomatosis [5]. Diagnostic challenges such as culture-negativity and the poor sensitivity of blood PCR lead to missed and delayed diagnoses [5]. While less common in the present-day, epidemic typhus causes a febrile illness with mortality rates reaching 60% in the pre-antibiotic era [6]. A concerning feature of epidemic typhus is its ability to reactivate decades after initial infection in the form of Brill-Zinsser disease [6]. Brill-Zinsser disease may trigger subsequent typhus epidemics when body lice infestation is present. Lastly, louse-borne relapsing fever was widespread worldwide until the early 20th century but is now largely confined to the Horn of Africa and among refugees originating from this region [7].

## Scabies

Unlike body lice, scabies mites are not vectors for pathogen transmission [1]. Scabies is a contagious skin disorder caused by the burrowing of the *Sarcoptes scabiei var. hominis* mite into the upper layer of the skin [1]. Scabies causes intense pruritus due to a delayed hypersensitivity reaction, triggering significant psycho-social distress. Scabies lesions may also act as a portal of entry for secondary bacterial infections, which may be associated with rheumatic fever and post-streptococcal glomerulonephritis [1]. Scabies is primarily transmitted through prolonged skin-to-skin contact with an infested individual. A more severe form of scabies, known as crusted (or Norwegian) scabies, occurs in immunocompromised individuals and is characterized by a significantly higher mite burden, requiring distinct management approaches [1].

## Body lice and scabies co-infestation

While scabies and body lice infestation share risk factors, co-infestation with both ectoparasites has been rarely described [1]. One of the few studies addressing this issue, conducted among homeless populations in France, concluded that the two diseases are associated with distinct subpopulations [8]. The few other studies that simultaneously assess scabies and pediculosis are focused on head lice rather than body lice [9]. In a proof of principle study in the Solomon Islands, a trial of ivermectin mass-drug administration for scabies reduced the burden of head louse infestation, but body lice infestation was not assessed [10]. Despite the paucity of data, another source suggests that poly-ectoparasitosis is frequent in resource-poor communities [11].

## MSF-Safai program

The MSF-Safai program suggests that body lice and scabies co-infestation may be more common than previously documented. In 2016, the overcrowded and unhygienic living conditions of migrants, refugees, and asylum seekers in Serbia accelerated body lice and scabies transmission. At that time, an estimated 7,720 migrants were living in the country, of which 1,200 were without shelter (sleeping rough) [2]. The closure of various European borders in early 2016 led to an increased length of stay in Serbia for migrant populations moving along the Balkan route [2,3]. As a consequence, many migrants, refugees, and asylum seekers in Serbia were living in overcrowded barracks near Belgrade’s former train station [2]. The MSF-Safai clinic was located 500 meters from the barracks and recorded the numbers of individuals presenting with dermatologic symptoms. From August 2016 until March 2017, ~2830 cases of body lice and 1595 cases of scabies were documented, with a peak of both infestations between December and February (S1 Appendix). For these aggregate statistics, rates of re-infestation and co-infestation were not documented. Nevertheless, dedicated screening programs conducted in the barracks commonly identified co-infestation. Among 563 individuals residing in the barracks, 82% (*n* = 464) were diagnosed with ectoparasitosis: 36% (*n* = 165) with body lice infestation only, 33% (*n* = 151) with scabies only, and 32% (*n* = 148) with body lice and scabies co-infestation [2]. In February 2017, over 300 cases of body lice and scabies co-infestation were diagnosed in a single week [2]. In April 2017, co-infestation remained common: of 982 screened ectoparasitosis cases, 480 (49%) were body lice, 360 (37%) were scabies, and 142 (14%) were co-infestations [2]. Increased rates of body lice infestation were recorded after systematic screening the clothing of underhoused migrants. Body lice arthropods and eggs were visible in migrants’ clothing (S1 Appendix). A presumptive diagnosis of scabies was made clinically, based on signs and symptoms, as microscopy and dermoscopy were unavailable [12]. Given the absence of imaging devices, scabies may have been under- or over-diagnosed. Similarly, body lice detection was limited to inspection of clothing seams, which may undercount low-burden infestations.

To control ectoparasitoses, MSF-Safai implemented a multidisciplinary strategy involving medical, environmental health, and vector-control teams. Measures included delousing, showers, laundry access, distribution of clean clothing and blankets, and treatment of infested items with insecticide in hermetically sealed plastic bags (Table 1). In total, 1,160 blankets were collected for washing, and 966 were sprayed with 20% permethrin. Approximately 1900 permethrin-impregnated clothing and blanket kits (0.5 grams active ingredient/m^2^) were air-dried before distribution. Because permethrin cream and oral ivermectin were unavailable in Serbia, alternative treatments were used. Body lice infestations were managed with a topical spray containing 0.19% permethrin once (with synergistic piperonyl butoxide), along with permethrin-impregnated clothing [13]. Scabies cases were treated once with benzyl benzoate 25% lotion applied to the entire body after a shower and rinsed after 24 hours—requiring prolonged unclothed contact and thus posing logistical challenges in settings without adequate privacy. Inter-cultural mediators promoted awareness using multilingual visual materials. Screening for louse-borne diseases did not occur, nor did contact tracing. Despite resource constraints, these coordinated interventions likely contributed to reducing ectoparasite transmission, though long-term longitudinal data are missing.

Oral ivermectin (200 µg/kg, two or three doses 7–14 days apart) offers an effective and potentially simpler alternative for both scabies and body lice infestation, but accessibility is limited in many jurisdictions despite its status as an essential medicine [10,14]. Repeating treatment is challenging for mobile populations whilst individuals with *Loa loa* hyper-filaremia risk encephalopathy.

## Water and sanitation as a human right

The human right to water and sanitation is recognized by the United Nations General Assembly as part of binding international law (resolution 64/292 in 2010, reaffirmed by resolutions 74/141 in 2019 and 76/153 in 2022) [15]. State governments thus act as duty-bearers in the provision of sanitation services. Furthermore, member countries of the European Commission have committed to the so-called AAAAQ criteria, assuring the availability, accessibility, affordability, acceptability, and quality of sanitation services. Epidemics of poly-ectoparasitosis demonstrate that water remains inaccessible for migrants, refugees, and asylum seekers experiencing homelessness.

## Conclusion

By systematically screening unsheltered migrants, refugees, and asylum seekers for body lice and scabies infestations, MSF-Safai identified thousands of cases of poly-ectoparasitosis. Body lice and scabies co-infestation is a poorly described entity that reflects a neglected area of research and may cause outbreaks of fatal disease. In situations where the human right to water and sanitation isn’t respected, public health programs should systematically screen for different forms of ectoparasitosis and provide ectoparasite control to prevent morbidity and mortality. Programs should incorporate simple, syndromic registers for scabies, body lice, and co-infestations, and—where feasible—entomological sampling of lice as sentinels for *B. quintana* and other louse-borne pathogens. The exclusion of unsheltered migrant populations from water and sanitation constitutes a violation of human rights and poses significant public health risks. The MSF-Safai program illustrates the necessity of alternative approaches where standard treatments and safe living conditions are inaccessible. In such contexts, ectoparasite screening alone is necessary albeit insufficient; advocacy for equitable water access is both a scientific and ethical imperative.

**Key learning points**

1) During scabies outbreaks, systematically screening for body lice infestations may identify considerable numbers of concealed pediculosis corporis cases.
2) Multidisciplinary coordination involving environmental health officers, vector-control experts, and medical professionals is necessary to effectively control outbreaks of scabies-body lice poly-ectoparasitosis.
3) Improving access to water, sanitation, and hygiene, including showers and laundry services, is necessary to curb ongoing ectoparasite transmission.
4) Oral agents such as ivermectin may be more feasible and acceptable than many topical agents in settings with limited privacy and water access.

## Supporting information

S1 Appendix Pictures of body lice and additional pictures of the barracks environment. (PDF)
