# Supplementary figures and images for: Body lice and scabies co-infestation among unsheltered migrants, refugees, and asylum seekers and the right to water and sanitation
Source: PLoS Negl Trop Dis. 2025 Dec 10;19(12):e0013807. doi: 10.1371/journal.pntd.0013807 (PMC12694801; doi:10.1371/journal.pntd.0013807)

**Appendix 1: Pictures of body lice and additional pictures of the barracks environment**

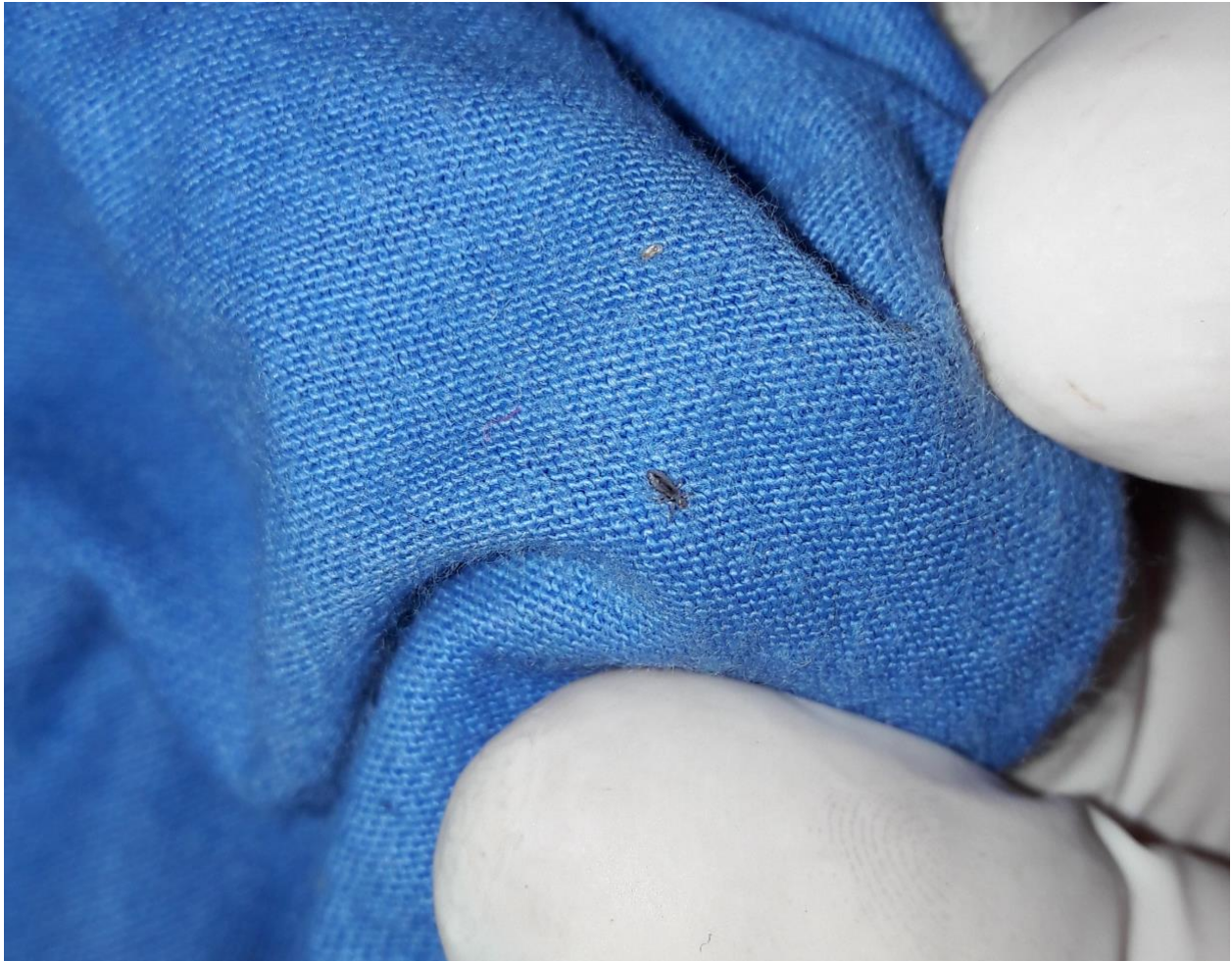

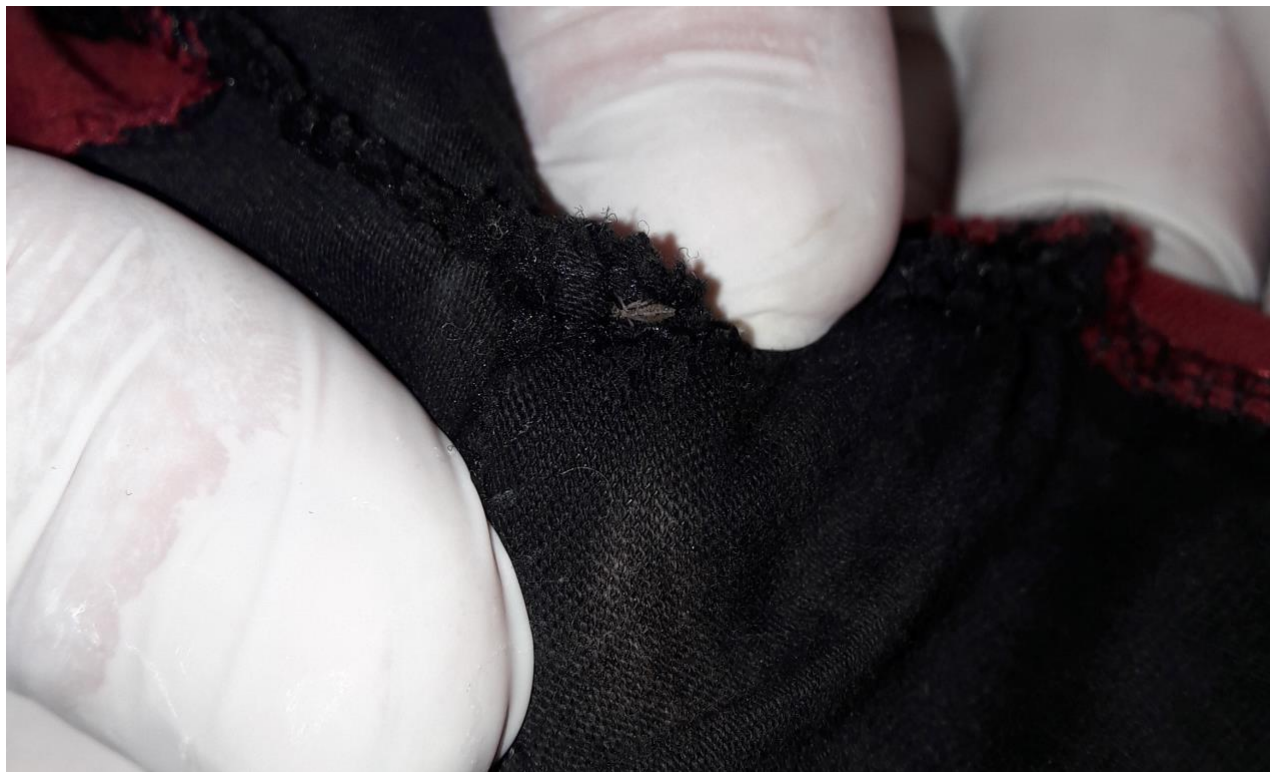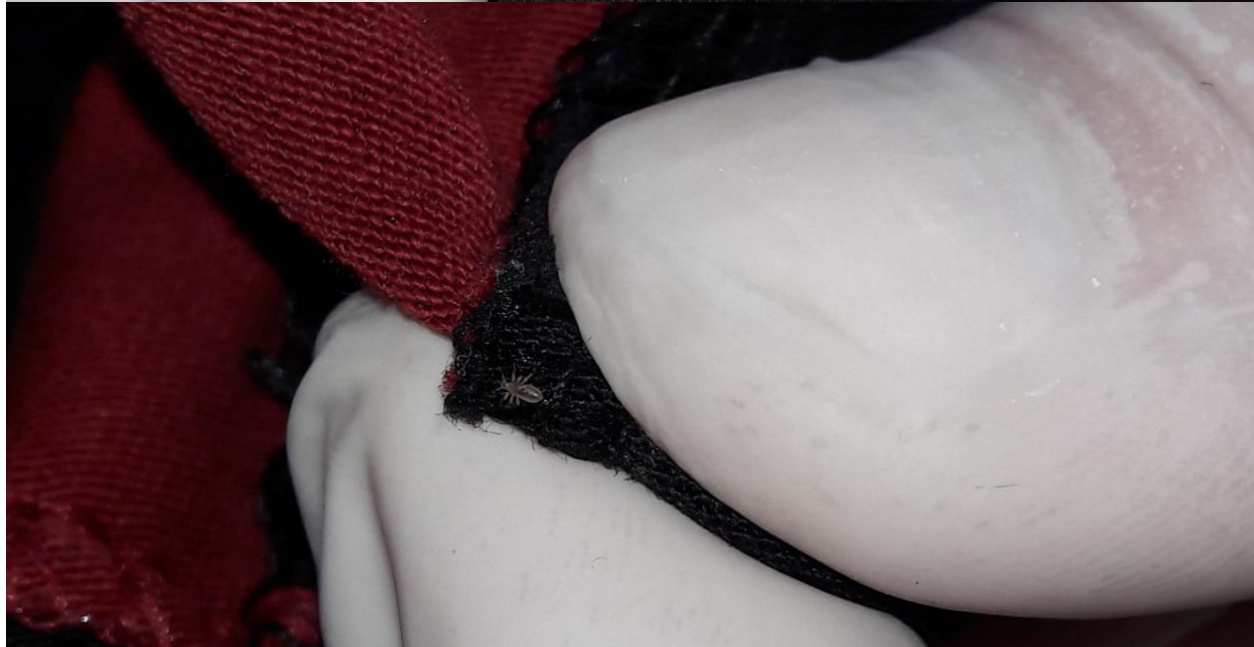

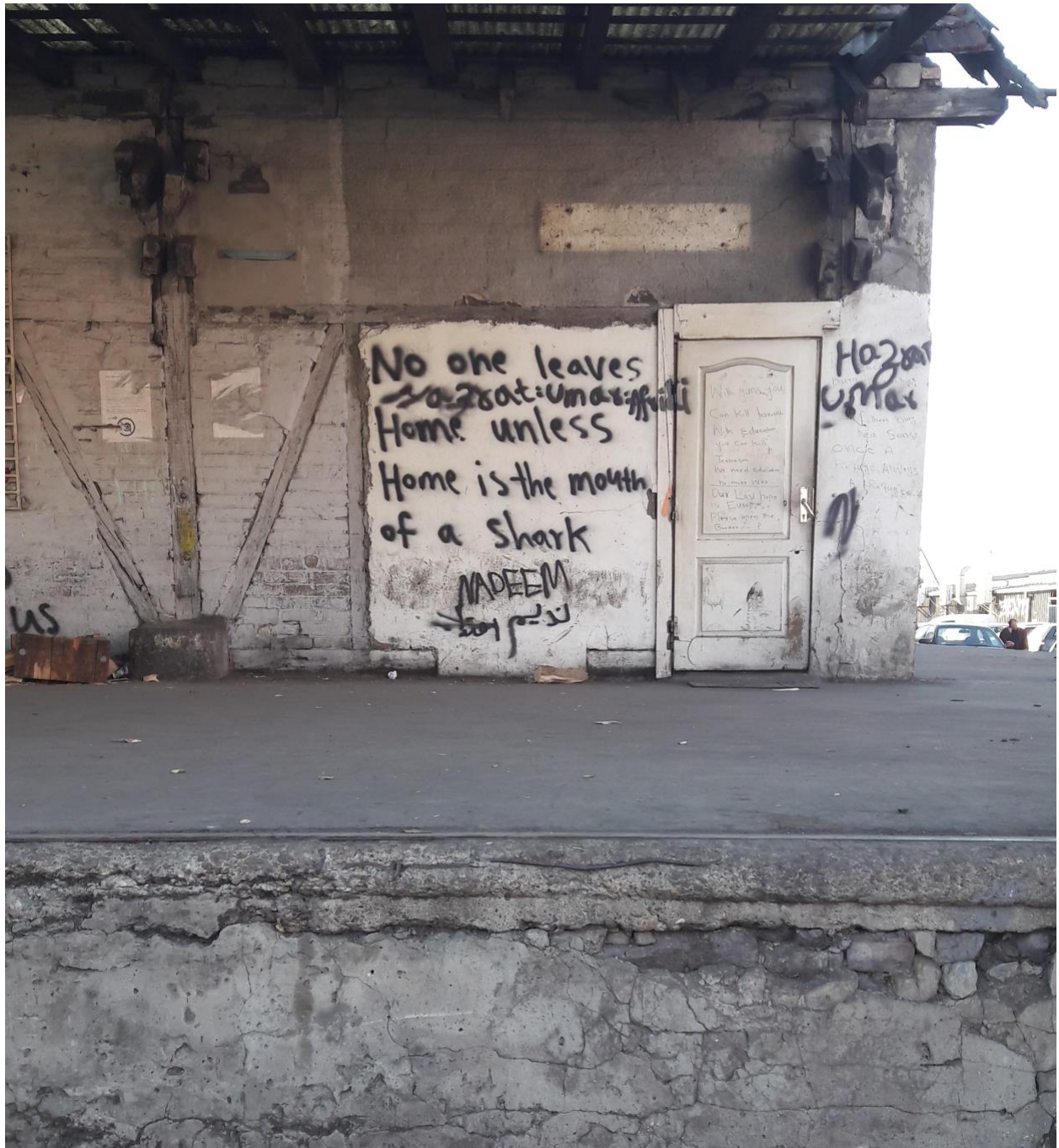

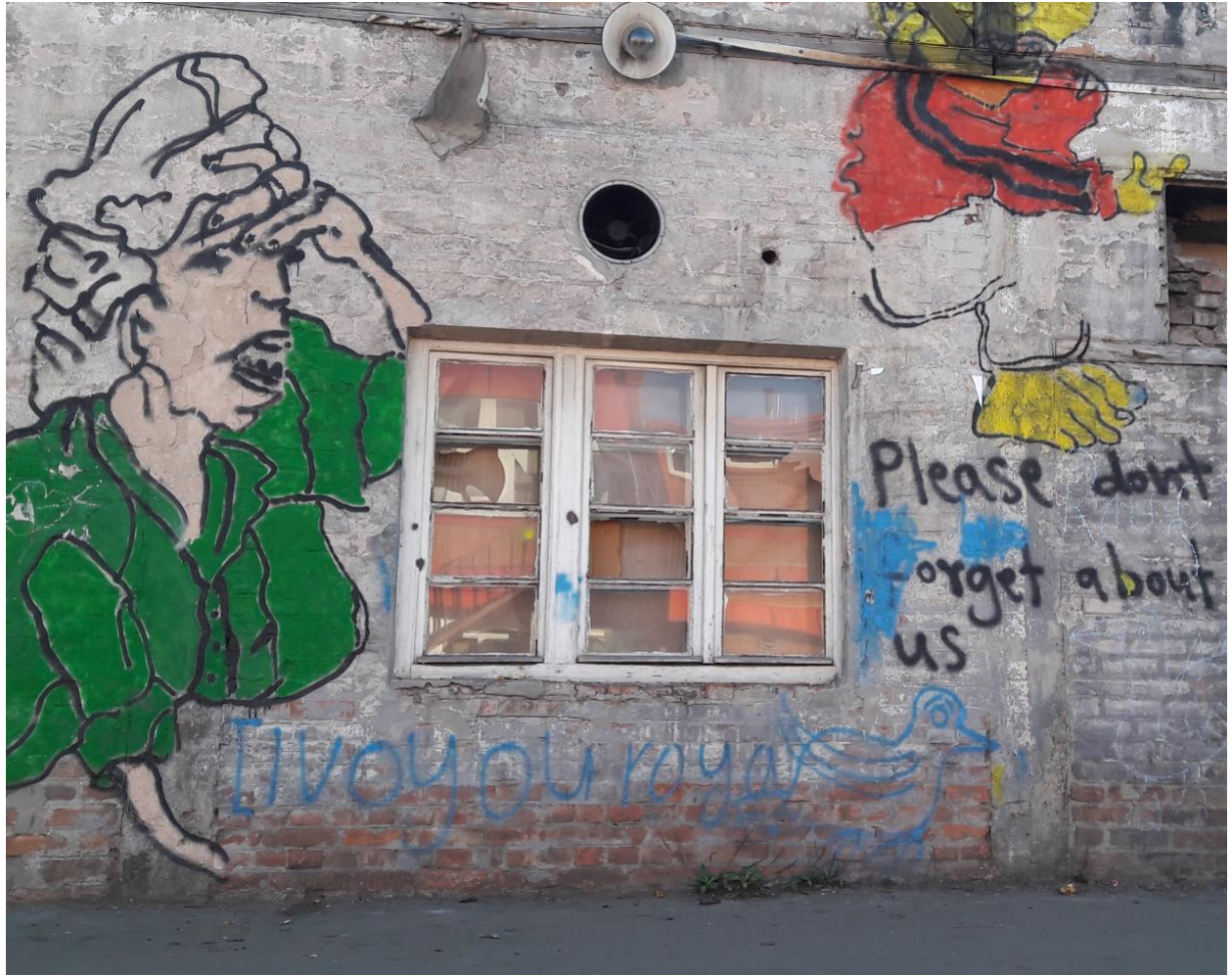

Supplement: S1 Appendix — (PDF) [file pntd.0013807.s001.pdf]
